# Spheroid-Based Tissue Engineering Strategies for Regeneration of the Intervertebral Disc

**DOI:** 10.3390/ijms23052530

**Published:** 2022-02-25

**Authors:** Jesil Kasamkattil, Anna Gryadunova, Ivan Martin, Andrea Barbero, Stefan Schären, Olga Krupkova, Arne Mehrkens

**Affiliations:** 1 Spine Surgery, University Hospital Basel, Spitalstrasse 21, 4031 Basel, Switzerland; jesil.kasamkattil@usb.ch (J.K.); anna.gryadunova@unibas.ch (A.G.); stefan.schaeren@usb.ch (S.S.); arne.mehrkens@usb.ch (A.M.); 2 Department of Biomedicine, University Hospital Basel, University of Basel, Hebelstrasse 20, 4031 Basel, Switzerland; ivan.martin@usb.ch (I.M.); andrea.barbero@usb.ch (A.B.); 3 World-Class Research Center “Digital Biodesign and Personalized Healthcare”, Sechenov First Moscow State Medical University, 119435 Moscow, Russia; 4 Lepage Research Institute, University of Prešov, 17. Novembra 1, 081 16 Prešov, Slovakia

**Keywords:** nucleus pulposus, annulus fibrosus, cell therapy, regenerative medicine, intervertebral disc degeneration

## Abstract

Degenerative disc disease, a painful pathology of the intervertebral disc (IVD), often causes disability and reduces quality of life. Although regenerative cell-based strategies have shown promise in clinical trials, none have been widely adopted clinically. Recent developments demonstrated that spheroid-based approaches might help overcome challenges associated with cell-based IVD therapies. Spheroids are three-dimensional multicellular aggregates with architecture that enables the cells to differentiate and synthesize endogenous ECM, promotes cell-ECM interactions, enhances adhesion, and protects cells from harsh conditions. Spheroids could be applied in the IVD both in scaffold-free and scaffold-based configurations, possibly providing advantages over cell suspensions. This review highlights areas of future research in spheroid-based regeneration of nucleus pulposus (NP) and annulus fibrosus (AF). We also discuss cell sources and methods for spheroid fabrication and characterization, mechanisms related to spheroid fusion, as well as enhancement of spheroid performance in the context of the IVD microenvironment.

## 1. Introduction

A long-lasting episode of low back pain (LBP) affects 80% of people at least once in their lifetime. The major cause of LBP is degenerative disc disease (DDD), an age-related pathology of the intervertebral disc (IVD) [1]. During DDD, the affected IVD suffers from progressive loss of cells and the functional extracellular matrix (ECM), leading to spinal instability and the development of chronic pain [1]. Although discogenic LBP significantly affects the quality of life by causing disability, practically no effective treatments exist. A surgical standard of care for DDD is spine fusion, which permanently connects two or more vertebrae in the spine. Spine fusion carries risks of slow recovery and adverse effects, e.g., accelerated degeneration of adjacent IVDs or implant instability, to name a few [2,3]. In fact, ~20% of spine fusion surgeries fail to improve symptoms (long-term pain) for various reasons [4,5].

Emerging regenerative IVD therapies (recently reviewed in [6,7]) envision the minimally invasive application of autologous cells that are expected to support the IVD by differentiating into IVD-like cells and/or by secreting trophic and anti-inflammatory factors [7,8,9,10]. Usually, cells are injected in suspension or combined with a biocompatible material, to provide initial mechanical stability and protection after implantation. However, despite substantial advances, none of these strategies have been widely adopted clinically. The challenges for IVD cell therapies are related to the unique anatomical structure, biochemical composition, and microenvironment of human IVD [7,8,10]. Numerous publications have suggested that therapeutic cells may suffer due to lack of nutrients and oxygen and high levels of lactate, inflammation, and complex (often non-physiological) loading [8,11,12,13,14,15].

Recent developments in other fields of tissue engineering demonstrate that spheroid-based approaches could help overcome some of these challenges due to their superior regenerative performance and/or resistance when compared to single cells [16,17]. Spheroids are self-assembling living microtissues based on multicellular aggregates that mature by the formation of intercellular contacts on non-adhesive substrates [18,19,20,21]. Their 3D architecture controls cell phenotype and function and enables cells within spheroids to favorably respond to loading. It is believed that 3D configuration provides spheroids with a higher ability to adhere and protects cells from harsh conditions [22,23]. However, the size and cell density of spheroids should be precisely controlled due to the possibility of necrotic core formation resulting from impaired fluid exchange, as shown in spheroids with a size above 500 µm in diameter [24]. Spheroids have the intrinsic capacity to fuse, which allows for structural and functional integration of spheroid-based grafts. Spheroids have been used in combination with biomaterials or alone as scaffold-free products [17]. Spheroid-based technology is in clinical use for articular cartilage repair and under investigation for regeneration of skin, blood vessels, and other tissues, supporting the feasibility and translational potential of this strategy for IVD regeneration [17,25].

Alone or in combination with injectable biomaterials, spheroids represent a promising, minimally invasive IVD treatment. Although the concept of spheroids is not new, less attention has been given to testing and reviewing this strategy in the IVD field. The main goal of this review is to outline our perspective on possible developments in spheroid-based tissue engineering of the main IVD tissues: nucleus pulposus (NP) and annulus fibrosus (AF) (Table 1)**.** We also provide a brief overview of cell sources and methods for spheroid fabrication and characterization, describe mechanisms underlying spheroid fusion/integration with target tissues, and discuss the enhancement of spheroid functions in the context of the IVD microenvironment.

## 2. Cell Sources for IVD Tissue Engineering

Cell sources for IVD repair were recently reviewed [33,34,35,36]. Autologous IVD cells are commonly isolated from specimens removed during surgeries, expanded, and transplanted back to augment IVD cell population. NP and AF cells showed promise in preclinical settings and clinical trials, leading to the emergence of clinical products that support autologous cell supplementation [37,38,39,40]. Although these cells are likely affected by pre-existing degeneration [41,42], they are able to survive in an IVD-specific microenvironment. Unlike most other cell types, NP cells constitutively express hypoxia-inducible factor 1-alpha (HIF1α), which makes them well equipped to manage the limited nutrient supply to a certain extent [39,43]. However, autologous IVD (especially NP) cells are very scarce in adults, and their phenotype changes with aging [44]. The slow expansion rate and the loss of native phenotypic features during the expansion in the monolayer are also potential drawbacks [45]. Multipotent tissue-specific NP progenitor cells (NPPC) were recently identified and investigated for their enhanced regenerative capacity [46,47,48]. NPPCs are characterized by a spheroid colony forming (in contrast to fibroblastic colony forming) and positivity for the cell surface marker angiopoietin-1 receptor (Tie2) [46,47]. However, it has been a challenge to implement NPPC in clinics due to their extremely low yield and the fact that their numbers decrease with age and degeneration [46,47], but not as drastically as notochordal cells—a unique sub-population with stem cell properties originating from embryonic notochord [33]. The transition of notochordal cells into chondrocyte-like cells before adulthood, and the subsequent decrease in proteoglycan content, is believed to cause NP degeneration [49].

**Figure 1:**
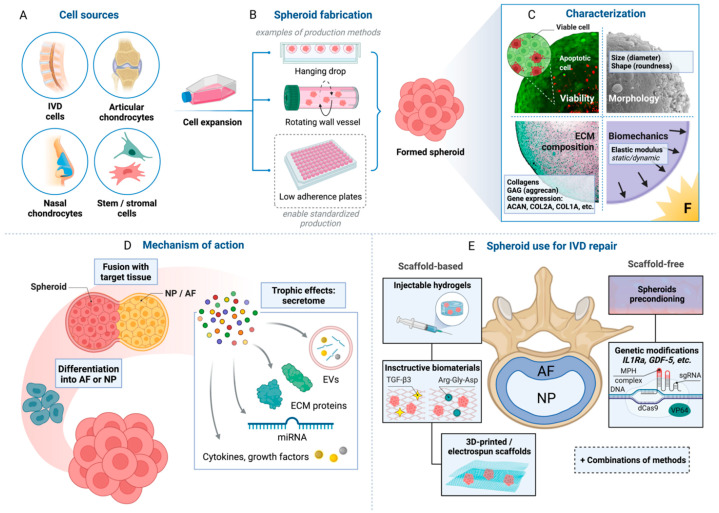
Spheroid-based regeneration of the intervertebral disc (IVD). (**A**) Several cell sources have been proposed for IVD repair. (**B**) While a variety of spheroid fabrication methods are suitable for research purposes, low adherence plates facilitating large-scale standardized spheroid production might be the first-choice technology for clinical application. (**C**) The systematic characterization of the produced spheroids (e.g., cell viability, spheroid geometry, extracellular matrix (ECM) composition, and biomechanical properties) is essential to ensure their applicability for IVD repair. (**D**) Spheroids can exhibit several modes of action to support the IVD, including rapid fusion with target tissue and mechanical support, differentiation of the constituent cells into IVD-like cells, and by secretion of biomolecules (cytokines, growth factors, extracellular vesicles (EVs), ECM proteins, etc.). (**E**) Both scaffold-based and scaffold-free approaches demonstrate the potential for the regeneration of the IVD. IVD = intervertebral disc, ECM = extracellular matrix, GAG = glycosaminoglycans, NP = nucleus pulposus, AF = annulus fibrosus, EVs = extracellular vesicles. Created with BioRender.com.

Given the limited availability of healthy autologous IVD cells, there has been an interest in other cell types (Figure 1A). Induced pluripotent stem cells (iPS) emerged as an adult cell source with great potential to address existing limitations of sparsity and donor site morbidity. Although iPSs still bear the concern of teratoma formation and uncertain differentiation capability, it is possible to avoid these features by pre-differentiation into committed cell lineages, e.g., notochordal cells, as recently demonstrated by regenerative effects in vivo in a porcine model [50].

To date, the most extensively investigated cell type for IVD repair has been adult mesenchymal stem/stromal cells (MSCs) [45,51], e.g., bone marrow-derived [52,53], adipose tissue-derived [54], or muscle-derived [55]. Implantation of MSCs has resulted in the restoration of the IVD height, IVD-like phenotype expression, ECM synthesis, and improvement in MRI signals [42,51,56]. An ideal therapeutic cell type should differentiate toward NP/AF cell-like phenotypes in vivo. A number of NP and AF phenotypic markers have been identified (e.g., Brachyury, FOXF1, PAX1, and KRT19) and MSCs showed good differentiation capacity towards IVD cell types in an IVD-specific microenvironment [33]. Notably, MSCs are effective at repairing large lesions (6 × 20 mm) in the partial-thickness AF-injury sheep model [56] with the recovery of disc height and functional biomechanical properties of repaired IVD tissues [57,58]. Although promising outcomes were found in animal and pilot clinical trials [59], MSC-based IVD therapy is not yet in routine clinical use [6,60,61], possibly due to variable differentiation capacity leading to inferior constructs [62] and/or the fact that these cells are not performing as expected in a harsh, degenerated microenvironment, which negatively affects their survival, function, and differentiation [8,62,63,64,65,66].

It has been demonstrated that adult chondrocytes from other locations may represent a better alternative in terms of resilience in harsh microenvironments [15,67]. In contrast to MSCs, articular chondrocytes (AC) (particularly from younger donors) show an enhanced ability to survive and produce ECM in porcine IVD defects in vivo [68]. However, chondrocytes might retain their phenotype and produce ECM with cartilage-like composition, possibly affecting the biomechanical properties of the IVD in vivo [33]. Although autologous AC are clinically used for IVD repair [6], they might also be influenced by pre-existing degeneration and donor site morbidity remains an issue. Notably, cells derived from other locations, namely nasoseptal chondrocytes (NC) originating from hyaline cartilage of the nasal septum, demonstrated superior ability over MSCs and AC to survive in osteoarthritic and DDD conditions but their ability to form functional ECM still remains impaired [69,70]. NC recently emerged as a promising cell source for cartilage pathologies. NC exhibit features of self-renewal and adapt to heterotopic transplantation sites, including osteoarthritic knee cartilage defects [71,72,73]. Engineered NC cartilage grafts are already being used to repair cartilage defects in patients [74,75]. Neuroectoderm-derived NC can be distinguished from mesoderm-derived AC by lack of expression of specific HOX genes and reprogrammed to stably express HOX genes typical for AC, as shown in goat articular cartilage defects [71]. In view of the IVD, adaptation of NC to NP/AF phenotypic features remains to be investigated.

### Spheroid Configuration of Therapeutic Cells

Spheroids can be derived from somatic as well as stem/stromal cells. In IVD repair, spheroid cultures could reduce the rate of dedifferentiation and enable rapid reimplantation of autologous NP/AF cells. Recently, spheroid-formation assay was successfully applied to the expansion of NPPC. Zhang (2020) demonstrated that this culture significantly enriched the percentage of Tie2-positive NP progenitors (vs. monolayer), possibly bringing NPPC closer to clinical application [76]. Spheroid configurations were also shown to improve the ability of MSCs to differentiate into chondrogenic and osteogenic lineages [64]. Furthermore, human MSCs spheroids performed better than single cells and differentiated into NP-like cells scaffold-free [77,78] and in hydrogels [79]. Similarly, iPS were shown to form microtissues differentiating into NP-like cells [80], with the capacity to be implanted (or injected) into the IVD. Notably, autologous AC-based spheroids were extensively investigated in preclinical settings and then implemented in patients suffering from traumatic or degenerative injuries of articular cartilage, leading to clinical benefit [81,82,83]. Compared to human AC spheroids and/or monolayer, NC spheroids manifest higher viability, more uniform morphology, and higher expression of COL2A1 and SOX9 [84]. Spheroids generated from human nasoseptal cartilage progenitors (i.e., a subpopulation of chondrocytes derived from superficial zone of human nasal septal cartilage samples shown to display progenitor cell features [85,86]) exhibit increasing biomechanical stability, synthesis of TGF-β1/2/3, and enhanced expression of ECM components over time even without a chondrogenic stimulus (compared to monolayer) [84]. Moreover, our group has recently demonstrated that NC spheroids (NCS) not only accumulate ECM without chondrogenic stimuli but also survive and fuse with NP spheroids in the DDD-mimicking microenvironment and are compatible with spinal needles for minimally-invasive application [87]. The potential use of spheroid-based regeneration methods for IVD repair is depicted in Figure 1.

## 3. Spheroid Formation and Characterization

### 3.1. Mechanisms of Spheroid Formation

In the first stage during chondrogenesis, known as pre-cartilage condensation, MSCs aggregate, increase cell density, and upregulate their hyaluronidase activity [88]. Consequently, MSCs stop proliferating, express ECM-related adhesion molecules, slow down the cell movement due to decreased hyaluronan in the ECM and promote close cell-cell interactions, which triggers signal transduction pathways involved in chondrogenic differentiation [89]. Following similar principles, three crucial steps are involved in the spheroid formation. First, dispersed cells begin to aggregate through establishing loose integrin-ECM bonds followed by upregulated cadherin expression and accumulation due to cell-cell contact. Consequently, compact spheroids form due to the homophilic cadherin-cadherin interactions [90]. The classic cadherins represent calcium-dependent, homophilic, cell-cell adhesion molecules found in nearly all solid tissues [91]. Prior studies have noted the importance of cadherins in both spheroid formation and fusion [92,93]. The spheroidal organization of the cells induces the synthesis of endogenous ECM which can affect the cell behavior in various ways. The cell-ECM interaction through receptor-mediated signaling might directly regulate the cell functions or ECM-associated growth factors can control the cell proliferation and differentiation thus regulate the cell phenotype [94]. For example, integrins were demonstrated to physically bridge the ECM to the network of cytoplasmic actin microfilaments providing an appropriate molecular scaffold for signaling components, resulting in changes of cell shape and actively regulating cell proliferation and differentiation [95]. Moreover, the integrins were proposed to act as direct mechanotransducers controlling many aspects of cell function including cytoskeleton remodeling and migration through physical forces [96]. On the other hand, the ECM proteins can regulate the growth factor bioavailability by establishing stable gradients of growth factors such as FGFs, HGF, and VEGFs [97]. They might be bound by ECM-associated proteins like fibronectin, by collagens and proteoglycans, or in combination with heparin and heparin sulphate [98]. This could lead to the activation of MAPK/ERK pathway resulting in modifications of intracellular signaling [99], which has already been described to play a key role in chondrogenesis [100]. Recently, it has been shown that increasing the actomyosin contractility could protect NP cells from TNFα-induced ECM loss thus demonstrating the relation between inflammation triggered ECM loss and mechanotransduction [101]. Therefore, the accumulation of ECM in spheroids could potentially support the cells to regulate their biophysical properties thus instruct them to cope in the harsh IVD microenvironment. The ECM also acts as a physical barrier that could protect the cells within the inner layer of the spheroid from direct exposure to harsh conditions such as inflammation or acidity [102]. Furthermore, since the self-assembly of the cells to a spheroid increases cell adhesion properties [18], the potential of the spheroids to reside within the disc after injection increases preventing cell leakage and with it associated problems such as osteophyte formation [103].

### 3.2. Spheroid Formation Methods

Standardized large-scale spheroid formation methods are essential to obtain cost-effective and uniform spheroids for research and clinical application. The spheroid formation must be compliant with good manufacturing practice (GMP) for advanced therapy medicinal products (ATMP) guidelines and must respect the specifications and release criteria defined in the investigational medicinal product dossier (IMPD) [104,105]. Sterility, viability, identity, purity and potency tests have to be included to ensure the safety and efficacy of the spheroids. Furthermore, valid release criteria have to be established early in the product development process to ensure smooth clinical translation. For example, it has been demonstrated that clinically relevant FBS substitutes such as human platelet lysates or autologous serum will have the same effect on the cells [106,107]. It is advisable for saving time and costs to check early whether the factors used for the formation of spheroids are translatable into the clinical setting and moreover, if there are corresponding GMP compliant substitutes available for human use.

Several methods are available for the fabrication of spheroids (Figure 1B), among others pellet culture, hanging drop, spinner culture, rotating wall vessel, microfluidics, magnetic levitation, and low-attachment surface culture (reviewed in [108,109]). These methods are suitable for basic research purposes but not all of them are relevant for clinical application, mostly because of the high labor intensity and cost, hindering large-scale production [108,110]. Low adherence plates prevent cell adhesion promoting cell aggregation and spheroid formation on a large scale [111,112] and ensure reproducible formation of spheroids with ~500 µm in size and the roundness score above 0.70–0.95, indicating compatibility with injectable IVD therapy [113,114,115]. A lower roundness score is typical for an ellipsoid shape and/or formation of satellites, which indicates lower reproducibility in the spheroid formation process [116,117] and might result in injectability issues.

### 3.3. Spheroid Characterization

During spheroid generation, cell-produced ECM will be accumulated, which allows to mimic in vivo spatial distribution and physiological microenvironment of the cells. Furthermore, cell-cell interactions and cell-ECM interactions will be favored inducing the secretion of differentiation factors [118]. The systematic characterization of the produced spheroids is essential to ensure the reproducibility of spheroids geometry (regular spherical shape) and their injectability into the damaged IVD using standard surgical equipment, i.e., 30G to 22G spinal needle [92]. 

The size of the spheroids should be smaller than the inner diameter of the needle to be used for the injection of the spheroids into the target tissue. For example, a clinically relevant spinal needle (22G, BD, 405149) for the injection of spheroids into the degenerating nucleus pulposus tissue has a diameter of 600 µm thus the spheroids should be <600 µm to lower the risk of being damaged during injection [87]. The size of the spheroids also influences their viability, whereas smaller ones show better viability than larger spheroids [119]. The metabolic waste removal and nutrition diffusion to the core of the spheroids are relative to their size, thus a larger spheroid size (>500 µm) could lead to a necrotic core formation [120,121]. The cell viability of the produced spheroids should be monitored closely, since both necrotic and apoptotic cell death may initiate and maintain the degenerative process of the IVD by the induction of matrix metalloproteinases, MMP-1 and MMP-13, among other factors [122] (Figure 1C). Appropriate extracellular matrix content (high proteoglycan to collagen ratio, an abundance of aggrecan and collagen type II for NP; collagen types I and II for AF) and biomechanical properties (elastic modulus close to that of the native NP or AF) are further requirements for cellular aggregates designed for application in IVD repair [123,124,125].

The most common methodologies for spheroid characterization include conventional morphometric analysis for size and shape quantification, assay staining protocols (e.g., calcein AM, ethidium homodimer, etc.) to measure cell viability, histological and immunohistochemical analysis to evaluate ECM composition and cell organization, atomic force microscopy and controlled compression to identify biomechanical properties, as well as more advanced mathematical modelling and computational simulation to predict spheroids’ post-implantation behavior [126,127,128,129].

A direct evaluation of the fusion kinetics in vitro is also advisable in order to simulate the integration of the implanted spheroids into the host tissue. When placed in close proximity to each other, spheroids merge over time to form one larger structure, providing a simplified model that can be easily quantified, e.g., to evaluate the influence of the DDD microenvironment on spheroid integration [93,130]. As an example, the angle between the chondrocyte spheroid and NP spheroid increases to a maximum of 180° in 96 h of their fusion; therefore the integration of therapeutic spheroids into the defect area in native NP could be expected to take ~4 days [87]. Interestingly, it has been recently shown that fusion culture has contributed to increased collagen type II synthesis and accumulation in human chondrocyte spheroids compared to single aggregates, thereby providing evidence of newly formed, self-made ECM typical for native hyaline cartilage [131]. Since NP shares a similar collagen phenotype to that of cartilage, further studies on spheroid fusion are of particular significance to IVD repair.

## 4. Spheroid Interactions with Target Environment

For long-term functionality, the structural and functional integration of the spheroids into the damaged tissue is essential. The spheroids should (i) adhere to the target site, (ii) loosen up their compact 3D organization, while surface cells migrate into the damaged area, and finally (iii) complete integration into the defect area and synthesize and secrete injured site-specific proteins to support the tissue repair [111].

Spheroids exhibit liquid-like behavior and undergo coalescence similarly to liquid droplets [132] as explained by the differential adhesion hypothesis (DAH), which claims that the cells rearrange in order to increase the number of cadherin adhesive bonds and reduce free energy [133]. The first stage of spheroid integration, or adhesion to the target site, relies upon various types of cell adhesion molecules; the cadherins, however, are crucial for this process [134,135]. Pre-cartilage condensation, a process of particular relevance to chondrogenesis, is mediated by cellular condensation through N-cadherins [136]. While the extracellular domain of N-cadherin forms interactions between opposing cells, the intracellular domain is anchored to the actin cytoskeleton by α-catenin, β-catenin, and other signaling molecules [137].

Migration of surface cells into the damaged area is a driving factor for the second integration stage. Previous studies have examined the assembly and fusion of spheroids containing various cell types [138,139], and cell migration during the fusion process has been shown to involve cytoskeletal dynamics [140]. The lipid bilayer of a cell membrane is draped over the actin cortex, and they deform simultaneously, being tightly bound to each other by anchoring proteins, such as ezrin, radixin, and moesin [141,142,143]. During the initial steps of fusion, the actin cytoskeleton produces multiple thin, finger-like protrusions that push into the cells on the adjacent damaged surface. The invasive protrusions, or filopodia, are generated through the formation of parallel actin bundles by actin polymerization. At the apex of the filopodia, the plasma membranes of the neighboring cells make contact when the cell–cell recognition molecules form an intercellular adhesion complex that eventually develops into the fusion pores [144]. Over time, this zone expands to form a stable contact region between cellular aggregates and the defect area. The realignment of the actin cytoskeleton is required for the fusion of spheroids produced from primary chondrocytes, and the disruption of microfilaments inhibits the process completely [145].

The complete integration into the defect area is characterized by the synthesis and secretion of site-specific proteins to support the tissue repair. When placed on the damaged articular cartilage, chondrocyte spheroids have shown to cover the entire surface of the degenerated cartilage within 3 weeks [21]. The cells not only migrate out of spheroids but also synthesize new ECM composed primarily of collagen type II and proteoglycans (PG) [146,147]. ECM in articular cartilage is reconstructed and remodeled upon spheroid implantation since the new chondrocytes replace matrix macromolecules lost through degradation [148]; the underlying molecular mechanisms, however, have yet to be investigated.

Understanding the role of biomolecules involved in spheroid integration into the IVD is a step towards fine-tuning the abovementioned interactions and accelerated healing of the damaged tissue. It has been recently shown that the physiological dynamic compression of 0.4 MPa up-regulates N-cadherin expression in NP cells compared to static compression [149,150]. Spheroid preconditioning under dynamic mechanical loading therefore appears to be an appealing option to enhance the integrative potential by improving their adherence to the target site. Since chondrocyte migration is impaired by inflammatory stress typical for DDD condition, anti-TNF-*α* bioactive molecules, such as etanercept and adalimumab as well as anti- IL1β drugs, such as anakinra, could be incorporated into microparticles and integrated into the chondrocyte spheroids or used as medium additives prior to implantation [151].

## 5. Spheroid-Based Cell Therapies for Degenerative Disc Disease

IVD contains distinct anatomical regions, namely the nucleus pulposus (NP), annulus fibrosus (AF), and cartilaginous endplates [152,153], which are all substantially different and unique structurally, mechanically, and biochemically, and present challenges for IVD tissue engineering. Ideally, an engineered construct should closely resemble the ECM architecture of the target tissue and rapidly integrate within a defect. Numerous studies have investigated the use of various cell-laden scaffolds and hydrogels. Despite strong efforts, scaffold-based approaches are still limited in terms of reduced cell viability, inconvenient manipulations, and unwanted degradation patterns. A less explored way to generate IVD-like neotissue is a scaffold-free, spheroid-based approach when therapeutic cells build their own support ECM from the beginning, which might enhance biomimetic functions and fasten the regulatory approval process in clinical translation. However, as spheroid ECM can be considered immature compared to native tissue, a combination of spheroids with biomaterials could be a practical alternative for the regeneration of NP and AF (Figure 1E).

### 5.1. Nucleus Pulposus

The NP is a hydrated structure predominantly composed of a loose network of highly hydrated proteoglycans (PG) and collagen type II, with PG/collagen ratio 26:1 in healthy IVD [154]. Notably, the microenvironment of degenerated NP contains low levels of oxygen and glucose, acidic pH, high osmolarity (relative to other tissues), and complex loading [12,155]. These harsh conditions were shown to induce a cellular catabolic shift that accelerates the degradation of ECM and negatively influences the function of therapeutic cells [13,14]. The catabolic shift is characterized by upregulation of pro-inflammatory cytokines and ECM degrading enzymes, as well as downregulation of inflammation antagonists and inhibitors [156,157]. The survival rate of therapeutic cells in the NP is also affected by reduced nutrient supply due to large IVD size and endplate calcification [152]. Altogether, these conditions limit the numbers of therapeutic cells to be used. Strategies to regenerate NP should consider the specific anatomy, limited diffusion rate, and harsh microenvironment while providing resistance to the compressional and torsional stresses within the spinal column [158]. An ideal therapy for NP regeneration would be liquid before application (injectable) and rapidly solidify and/or integrate upon injection to ensure correct distribution and retention in the NP [62,159].

The intrinsic ability of spheroids to rapidly fuse with target tissue is believed to be crucial for regeneration [160]. In order to prevent their extrusion from NP, the adhesion of spheroids and migration of surface cells into NP, followed by spheroid remodeling, must take place. Consequently, spheroids are expected to secrete an NP-like matrix into the defect cavity, leading to restoration of IVD height, gap filling, and biochemical integration of spheroid cells into the surrounding NP tissue [22,112]. 

In scaffold-free conditions, a supportive material is not used, thus there is no need to consider long-term effects of an implanted scaffold [161]. 3D configuration and increased paracrine effects of spheroids (compared to 2D) are thought to enhance the differentiation potential of therapeutic cells. The ability of spheroids to synthesize their own ECM results in the encapsulation of cells in native ECM, the composition of which is driven by the original cell type and culture conditions [87,160]. In the clinical repair of cartilage defects in the knee, AC-based spheroids (Spherox) generated a hyaline-like structure and showed the potential to synthesize an articular cartilage-specific matrix [22,112]. We have recently demonstrated that ECM and biomechanical properties of spheroids derived from human NC are tuneable by cell culture supplements, possibly to match properties of target tissue (NP) and that spheroids of less than 600 µm are injectable into an (bovine) IVD through a spinal needle, without their mechanical damage [87]. A self-produced ECM of spheroids is also believed to retain growth and trophic factors and constitute a physical barrier between harsh target tissue and therapeutic cells [16]. However, in scaffold-free tissue repair, the cell numbers required to maintain the same 3D architecture as constructs that are scaffold-based, are higher. Importantly, native NP tissue contains, proportionally, very low cell numbers compared to the amount of ECM; thus the use of a supportive biomaterial might be warranted to maintain the graft volume. 

A biomaterial could be present after spheroid fabrication or already at the stage of spheroid assembly. Hydrogels have several advantages for NP repair, such as a 3D structure that generates volume and promotes cell adhesion, migration, and integration. Natural materials, such as collagen, hyaluronan, chitosan, or fibrin, mimic an in vivo environment, as they bear similarities with the native ECM. For example, injectable colloidal gelatine hydrogels with encapsulated MSCs support the NP-like differentiation, reduce cell leakage, and improve the survival of therapeutic cells in a rabbit model [162]. On the other hand, synthetic polymers, such as poly(lactide) (PLA), poly(glycolide) (PGA), and poly(ε-caprolactone) (PCL), offer easier processing, tuneability of mechanical properties and degradation patterns, and low immunogenicity [163,164].

While the combination of spheroids with hydrogels has yet to be investigated in the IVD field, spheroid-based constructs have already been tested in cartilage repair. As an example, an alginate/hyaluronic acid (HA) hydrogel was used to embed MSC spheroids in bi-layered osteochondral implants that supported the functional regeneration of articular cartilage in sheep [165]. An encapsulation of spheroids in an injectable biomaterial might help to hold them in place, protect them further from unfavorable microenvironments, and instruct them towards differentiation [166,167]. It would be expected that a biomaterial will not impair the ability of spheroids to spread and fuse with NP tissue but rather modulate these functions. In cartilage repair, it was suggested that delayed spheroid spreading, achieved by the use of PLGA/chitosan (CS)-containing constructs, can provide superior chondrogenic effects in vitro and in vivo due to the fact that spheroid 3D architecture is preserved longer [168]. Whether delaying spheroid spreading/fusion by the use of a biomaterial would produce beneficial effects in NP repair has yet to be investigated. It should be noted that spheroids rely exclusively on diffusion to transport nutrients and eliminate waste, so their interior might start suffering from a lack of nutrients, oxygen, and excess waste products, if spreading is inhibited for longer periods. Nevertheless, these negative effects and consequent onset of necrosis could be partially regulated by spheroid size and the total number of therapeutic cells in the NP.

Recent developments expanded the possibilities for modulating spheroid spreading (and other parameters) by generating composite spheroids, with a biomaterial included already during spheroid fabrication. In adipose tissue engineering, composite multicellular spheroids formed by MSCs and synthetic biodegradable nanofilaments showed enhanced adipogenic potential compared to homotypic spheroids. It was also demonstrated that the size of these spheroids could be readily controlled with the integrated amount of nanofilaments. Moreover, the material part of the spheroids could be used to sustainably release bioactive drugs (e.g., GFs) in order to fine-tune the properties of target tissue [169]. Including biomaterials during spheroid fabrication process was also shown to influence spheroid roundness in ligament tissue engineering [170].

Combining spheroids with an injectable instructive biomaterial is an attractive possibility for the regeneration of the NP. In the future, it will be necessary to define the best biomaterial for spheroid encapsulation with regard to their fusion kinetics (with target NP tissue and with each other) and biomechanical stability.

### 5.2. Annulus Fibrosus

Approaches to regenerate NP are likely to have limited success without sufficient repair of the AF, i.e., the outer ring of the IVD. The AF is composed of circumferential layers of lamellae formed by closely arranged fibers of collagen type I. AF provides load-bearing function, tensile resistance, and adequate support to maintain NP pressure [28]. During IVD degeneration, non-physiological loading and catabolic shift reduce ECM turnover, leading to the development of microdamage, clefts, and tears in the AF [171]. Due to the loss of PG and inflammation-associated upregulation of specific growth factors (NGF, VEGF), nerves and vessels from adjacent tissues grow deeper into the IVD [172], which causes nerve irritations and aggravates pain [172,173]. Strategies to regenerate AF thus focus on filling structural defects and rapidly restoring physiological ECM structure and function (collagen lamellae) to support AF’s tensile resistance and prevent excess nerve ingrowth. Persistent AF defects increase the risk of recurrent IVD herniations, which then require reoperations [174].

Strategies to mechanically repair AF were developed (sutures, patches) but none of these techniques significantly altered annular healing in animal models nor demonstrated long-term benefits in clinical trials [175]. It is crucial that AF implants maintain adhesion to target tissue, especially under strain. Novel AF sealants have been generated and showed promising results [175,176,177,178]. Compared to acellular therapies, cell-based implants improve ECM deposition and organization in animal studies and show more successful AF remodeling in the long-term [178]. Nevertheless, neither biological/mechanical properties similar to AF tissue nor native-like ECM organization were fully reproduced to date [178]. Importantly, regeneration of inner AF has been a challenge, as current implants fail to fully bridge inner AF defects.

Due to their intrinsic ability to adhere, spheroids could serve as building blocks for a living AF patch that fuses to larger 3D structures in situ or before implantation. Spheroid-based architecture could achieve successful defect bridging and fix the implanted material in place [178], especially if combined with a biomaterial [16]. Recent advances make it possible for spheroids to be seeded into biomaterials during or after the fabrication process, immobilized on pre-fabricated scaffolds, or embedded between scaffold layers in a patterned manner, possibly achieving the typical lamellar structure of the AF [179]. Combining spheroids with injectable hydrogels could efficiently fill irregularly shaped defects in a minimally-invasive and rapid manner as well as instruct the behavior of therapeutic cells [180,181].

A sufficiently porous biomaterial is needed to seed spheroids randomly or into a specific structure. Although spheroids were not widely explored in AF tissue engineering, recent developments expanded the possibilities for spheroid-biomaterial seeding in related areas of musculoskeletal repair. In bone tissue engineering, foaming/freeze-drying techniques were used to produce scaffold microporosity that promoted spheroid penetration into the scaffold and fixed them in place [16,17]. Spheroids were also generated in situ in a novel porous PLGA/CS scaffold obtained after lyophilization. ASC spheroids formed in these scaffolds promoted hyaline cartilage-specific chondrogenesis in vitro and structural/functional regeneration in vivo (rabbit model). This method reproducibly yielded spheroids of smaller sizes (diameter less than 200 μm), which facilitated the penetration of oxygen and nutrients into spheroids [182]. In situ generation of spheroids directly within an implantable scaffold might reduce culture time and lab manipulation, supporting the applicability in clinical AF repair. However, the exact pore size and porosity of scaffolds produced by methods like foaming or lyophilization might be difficult to control. 

3D printing can reproducibly control the internal pore size (50–800 μm), porosity, pore interconnectivity, and mechanical performance of tissue-engineered scaffolds. Huang (2013) used a solid freeform fabrication method to prepare PLGA-CS scaffolds that delayed spheroid spreading in cartilage repair. Their scaffold showed a fully interconnected macroporous structure and controlled geometry, maintained the 3D microenvironment of MSC spheroids, and a showed superior ability to regenerate chondral defects in a rabbit model when combined with spheroids (vs. single cells) [168]. 3D printing also holds promise to generate scaffolds that precisely fit the geometry of interest, allowing for guidance of the spheroid placement into specific shapes and geometries [179]. However, the automated seeding of spheroids onto 3D-printed scaffolds to produce a complex 3D construct has not yet been largely explored [16]. To precisely replicate AF structure using spheroids as building blocks, patterned micro- and nano-structures could be produced, e.g., by an innovative “lockyball” approach, where ASCs were immobilized into solid synthetic microscaffolds (lockyballs) fabricated by two-photon polymerization and designed with hooks and loops to enhance the retention and integration at the implantation site [183].

The generation of functional double-layered AF patches with one side promoting integration with inner AF and the other side sealing the defect from outside is an attractive proposition. In cartilage repair, Favreau (2020) developed compartmentalized, multi-layered implants seeded with spheroids to treat osteochondral defects. The first compartment was based on therapeutic collagen membranes associated with BMP-2 to provide structural support and promote subchondral bone regeneration, while the second compartment contained BMSC spheroids dispersed in alginate hydrogel to support the regeneration of the articular cartilage [165]. These implants showed promising results in a sheep model. With modifications relevant for AF tissue, a spheroid-laden part could be used to bridge AF tears while a cell-free layer could possibly serve for AF sealing.

In order to recapitulate natural ECM structure and facilitate interactions between living AF patches and resident cells, electrospinning could be the fabrication method of choice. Electrospinning and its modifications can generate randomly organized or aligned fibers that mimic the natural ECM and provide wide cell adhesion surfaces and adjustable porosity. This allows spheroid immobilization and modulation of spreading as well as cell migration and differentiation. An alignment of electrospun fibers was shown to regulate ASC spheroid functions. Non-aligned nanofibrillar structures demonstrated a heterogeneous dispersion of ASC spheroids, preventing efficient cell colonization of the nanofibers’ surface [184]. On the other hand, ASC spheroids seeded on aligned nanofibrillar structures (produced by jet-spraying) showed rapid and homogeneous cell dispersion, high viability, chondrogenic differentiation, and fused with each other, increasing the cell contact of the surface of the nanofibers. Therefore, fiber alignment that mimics the lamellar AF structure could produce a patch that integrates with target tissue more rapidly.

A combination of the above-mentioned fabrication methods may enhance the desired properties of (hybrid) living AF patches and/or immobilized spheroids in the scaffold. In skin tissue engineering, Lee (2020) seeded ASC spheroids onto a 3D-printed alginate-based mesh, which was followed by electrospinning of alginate/polyethylene oxide fibers directly onto the spheroids. The alginate scaffolding structure clearly retained the characteristics of the spheroids and maintained their superior regenerative capacity over scaffolds without the mesh [185].

The main function of spheroid-based living AF sealants would be to sustain tension generated by the NP and thus prevent NP extrusion until the defect is healed. Mechanical properties of AF sealants can be increased by crosslinking agents (genipin, glutaraldehyde, riboflavin), which also promote their attachment to native tissue. Genipin crosslinked (cell-free) hydrogels achieve biomechanical properties of AF tissue [186,187,188], even possibly outperforming FDA-approved materials under loading [188]. However, their failure to adhere to AF tissue at higher strains of 15–30% (typical for degenerative overloading) was also reported [186]. As some crosslinking agents might negatively influence cell viability, adhesion, and spreading, preliminary tests should be performed to select an appropriate agent/concentration for each material and application [186,187]. Nevertheless, recent advances in other fields of tissue engineering clearly demonstrate the potential of the synergistic scaffold-based and scaffold-free strategies for AF repair.

### 5.3. Enhancement of Spheroid Functions

Besides degradation of ECM, DDD is characterized by sterile tissue injury and unresolved inflammation. The evidence suggests that therapeutic cells can mediate tissue repair not only by differentiation towards target structures but also via the secretion of soluble factors that enhance tissue repair (Figure 1D). Directing therapeutic spheroids towards paracrine trophic, anabolic, and anti-inflammatory functions is an exciting strategy to potentiate their performance and resistance. It is known that spheroids already release higher amounts of growth factors and anti-inflammatory factors, compared to single cells [189,190,191]. The secreted biomolecules are entrapped in ECM and readily control a range of biological processes, becoming a source of relevant regenerative cues. Thanks to recent developments, the secretion of beneficial factors from spheroids can be further enhanced by specific 3D-culture conditions, providing superior functions even without the use of stimulative growth factors [189,192].

In addition to secretome, therapeutic properties of spheroids are mediated (at least partially) via exosomes, the nanometer-size type of extracellular vesicles (EVs) that carry RNAs, proteins and lipids from the parent cell [193,194]. Recently it was shown that inhalation treatment of lung spheroid-derived exosomes (as well as secretome) provided anti-inflammatory properties and improved lung regeneration in two animal models of pulmonary fibrosis [195]. The therapeutic potential of EVs in IVD regeneration was recently reviewed [196]. For example, MSC-derived EVs are believed to promote regeneration and proliferation and reduce inflammation and apoptosis in the IVD, possibly via miRNAs and other (yet unknown) mechanisms. The use of spheroids could improve the quality and possibly increase the yield of therapeutic vesicles. The application of spheroid conditioned medium containing therapeutic secretome and/or EVs could be considered as a cell-free alternative for the treatment of DDD, with a lower regulatory burden [190,191].

Tissue-specific functions were shown to be promoted by a biomimetic environment applied during spheroid generation in vitro. Biomimetic spheroid priming enhances their chondrogenic capacity and/or resistance in harsh conditions. In cartilage tissue engineering, scaffold-free chondrocyte spheroids generated under hypoxia, upregulated the expressions of collagen II and aggrecan at mRNA and protein levels, increased ECM deposition, and generated a higher quality of cartilage [83,197]. In IVD repair, preconditioning of MSCs with hypoxia is known to provide beneficial effects by activating a hypoxia-inducible factor (HIF) signaling pathway found to be involved in phenotype maintenance, metabolism, and homeostasis of the IVD [43]. It remains to be seen whether preconditioning of (MSC) spheroids with hypoxia (or other microenvironmental conditions) further augments their effects on IVD-specific phenotype and function.

Recently Muttigi et al. (2020) described the promising effect of spheroid priming with Matrilin-3, a noncollagenous ECM adaptor protein. Matrilin-3-primed ASC spheroids increased gene/protein expression of growth factors and reduced the secretion of hypertrophic ECM components. Furthermore, Matrilin-3-primed ASC spheroids induced the stable mRNA expression of SOX9, collagen type II, and aggrecan, and enhanced chondroitin sulphate accumulation in NP cells (indirect co-culture). Matrilin-3-primed ASC spheroids also facilitated IVD repair in a rabbit model with AF puncture-induced IVD degeneration [77], highlighting preconditioning as a useful approach to promote the regenerative capacity of spheroids for IVD repair.

Genetic modification could also enhance the chondrogenic capacity and resistance of spheroids. Genetic engineering to resist in a harsh IVD microenvironment has been widely considered [198,199]. Specifically, inflammation antagonists (e.g., IL1Ra) and IVD-related growth factors (e.g., GDF-5) appear to be promising targets [200]. Although not yet investigated in the IVD, genetically modified MSC spheroids with upregulated Runx2 were shown to overcome negative effects of a harsh microenvironment and promote regeneration in bone tissue engineering [201]. Major limitations of human genetic engineering are related to viral vectors and low (transient) expression of transgenes. Recently it was shown that the expression of non-viral transgenes could be maintained much longer in spheroids transplanted in vivo versus single cells. In hepatic regeneration, such a genetically modified spheroid system contributed to significantly higher therapeutic effects of transplanted hepatocytes in the host tissue [202]. Genetically modified spheroid systems might thus contribute to the maintenance of non-viral transgenes in the IVD and enrich anti-inflammatory and/or anabolic functions.

Instructive biomaterials providing physical and chemical signals required to modulate cellular behavior and reinforce particular spheroid phenotypes were designed [203]. Materials, e.g., with/without RGD peptides, would modulate spheroid spreading, while encapsulation of growth factors promotes spheroid fusion [165]. While not yet applied to spheroids, cell/growth factor-loaded particles ensured sustained release and chondrogenic differentiation of encapsulated therapeutic cells, aiding IVD regeneration in animal models [204,205]. Similarly, cell-free siRNA complexes encapsulated in injectable HA hydrogels retained release/activity over a prolonged period of time in vitro and in vivo [206]. Continuous supply of bioactive material combined with 3D cell configurations might enhance differentiation into chondrocyte-like NP cells as well as rejuvenate resident IVD tissue [204]. An implant combining MSC spheroids and a biomaterial with slowly released growth factors showed promise in sheep osteochondral repair [165].

Although single spheroids might lack in ECM organization being mechanically inferior to native tissue, spheroids are mechanosensitive, potentially enhancing the right interaction between an implant and target tissue upon loading [207,208].

## 6. Conclusions

In this review, we highlighted the potential of spheroid-based tissue engineering strategies for the repair and regeneration of the IVD. We first recapitulated the known aspects of spheroid tissue engineering and emphasized how promoting cell-ECM interactions in spheroids might be beneficial for IVD repair. We also introduced cell sources for IVD tissue engineering, with specific focus on 3D spheroid configurations, and enlisted fabrication and biomechanical/biochemical characterization methods to be used to reproducibly generate spheroids compatible with IVD tissue. Furthermore, mechanisms of spheroid integration into damaged tissues, including adhesion, migration of cells, and synthesis and secretion of site-specific proteins, were described to aid scientific understanding of underlying biological cues for tissue engineering purposes.

Spheroids can be applied in NP and AF both in scaffold-free and scaffold-based configurations, possibly providing advantages over cell suspension, as the 3D organization enables the cells to differentiate and synthesize endogenous ECM. In NP regeneration, spheroid-based strategies might prevent or delay a catabolic shift in therapeutic cells and/or accelerate graft integration in the DDD microenvironment. The use of scaffold-free spheroids might limit issues associated with biomaterials, such as inadequate degradation properties, thus simplify regulatory approval process. However, to generate volume within the NP tissue, the combination of spheroids with an injectable biomaterial should not be excluded from future investigations. In AF tissue engineering, spheroids could serve as building blocks for living AF patches, together with various biomaterials to seal the AF. A biomaterial could regulate the rate of spheroid fusion with AF, define spheroid position in a patch, and/or release factors that regulate spheroid functions. Spheroids’ capacity for rapid fusion might aid in filling deeper and irregular AF defects. The enhancement of spheroid functionality using a biomimetic and/or bio-instructive environment, as well as genetic modifications, are further measures to increase spheroid functionality in IVD regeneration.

From a clinical point of view, spheroid-based tissue engineering strategies for regeneration of the intervertebral disc represents an ideal minimal invasive treatment for a disease with a great burden for the patients and society in general. Unlike current surgical treatment options (most frequently fusion surgery) this kind of treatment could, once established, be performed as an outpatient procedure, which would further add to patient comfort. Ideally, this treatment will restore the function of the affected disc/motion segment and hopefully acceleration of adjacent segment degeneration (which often occurs after fusion surgery) will be avoided. In cases with more than one affected level, one could even treat several levels at the same time or add a level that may not seem “healthy” anymore but still seems to have sufficient function and may become symptomatic in the future. Adding such levels in the currently established treatment with fusion surgery is out of the question, as each added level would increase the potential for complications and adjacent segment degeneration.

## Figures and Tables

**Table 1:** Composition of healthy nucleus pulposus (NP) and annulus fibrosus (AF) and function of these components [26,27,28,29,30,31,32].

Tissue Function	Water Hydrostatic Pressure	Collagens Tensile Strength	PG Osmotic Pressure	Other Proteins Support of Matrix and Cells	Cells Homeostasis
**NP** (inner core, highly hydrated tissue)	70–90% *	15–20% ^‡^ mainly collagen type II	65% ^‡^	20–45% ^‡^	4000 cells/mm^3^
**AF** (outer IVD ring, elastic, and fibrous tissue)	60–90% *	50–70% ^‡^ mainly collagen type I	10–20% ^‡^	10% ^‡^	3000–9000 cells/mm^3^

* Percentage of wet weight. ^‡^ Percentage of dry weight of the IVD. PG = proteoglycans.
